# Advances in Electrical Discharge Machining of Insulating Ceramics

**DOI:** 10.3390/ma16175959

**Published:** 2023-08-30

**Authors:** Sergey N. Grigoriev, Marina A. Volosova, Anna A. Okunkova

**Affiliations:** Department of High-Efficiency Processing Technologies, Moscow State University of Technology STANKIN, Vadkovskiy per. 3A, 127994 Moscow, Russia; s.grigoriev@stankin.ru (S.N.G.); m.volosova@stankin.ru (M.A.V.)

**Keywords:** Al_2_O_3_, AlN, assisting means, electrical conductivity, electrical resistance, material removal rate, powder concentration, Si_3_N_4_, ZrO_2_

## Abstract

There are two main ways of carrying out the electrical discharge machining of the insulating ceramics: changing the electrical and chemical properties of ceramics due to additives in producing composites/nanocomposites and changing the electrical and chemical properties in the interelectrode gap. This review summarizes and analyzes the current data on the machinability in water suspension and hydrocarbons depending on the electrical properties of the ceramic composites and assisting means such as coating and powder. There are provided the existing approaches and original methods for solving the global problem of the electrical discharge machining of insulating ceramics, suggesting further development of the existing methods since, up to now, the experimental research is non-systemic. The dependencies of the machinability on the electrical properties of conductive ceramic composites, the specific electrical resistance of the assisting coating, and the assisting powder’s band gap and concentration for machining insulating ceramics are revealed. The higher the electrical conductivity, the higher the machinability of ceramic composites, and the lower the band gap, the higher the machinability for insulating ceramics. Two technological gaps were revealed in the powder’s concentration that can be a particular case of logarithmic decrement of attenuation. The proposed approach suggests using assisting powder with the lower band gap.

## 1. Introduction

Electrical discharge machining (EDM) can be successfully applied to single-phase ceramics, cermets, and ceramic/matrix composites as long as they exhibit electrical resistivity lower than 100–300 Ω·cm [1,2]. Non-conductive ceramics such as

**Figure 1:**
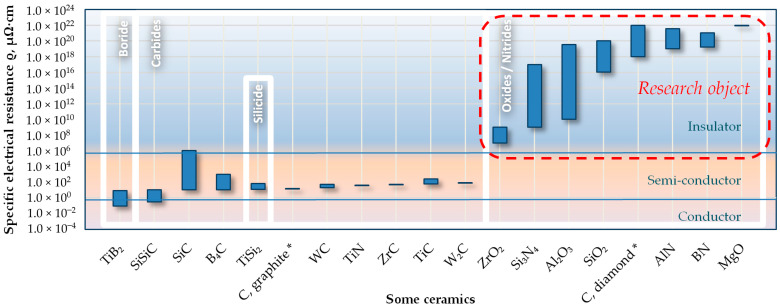
Specific electrical resistance ρ of some ceramics (* provided for reference).

- most of the oxide ceramics (alumina Al_2_O_3_, aluminum nitride AlN, magnesium oxide MgO, zirconia ZrO_2_);
- some nitride ceramics (silicon nitride Si_3_N_4_, boron nitride BN);
- oxide-nitride ceramics such as SiAlON, a solid solution of silicon nitride (Si_3_N_4_), where Si–N bonds are partly replaced with Al–N and Al–O bonds do not meet the minimum requirement of an electrical conductivity of 10^−2^ Ω·cm to be subjected to electrical erosion [3,4,5,6,7,8,9] (Figure 1). However, due to their exceptional exploitation properties, they are of tremendous industrial interest for some critical applications. Secondary electrically conductive phases are incorporated to influence such insulating ceramics’ electrical conductivity [10,11]. Metal-like carbides, carbonitrides, and nitrides of some transition metals that form predominantly metallic bonds (TiC, Ti(CN), TiN) serve the conductive phases in electro-conductive ceramic composites and nanocomposites based on oxide, nitride, or oxide–nitride insulating ceramics [12,13]. Metal-like carbides exhibit electrical conductivity similar to metals. They are delocalized metal bond *d*-element carbides based on iron, tungsten, tantalum, titan, vanadium, niobium, chrome, molybdenum, and nickel, of IV–X element groups, excluding platinum-group metals with a low electronegativity of 1.36–2.3 of Pauling’s scale and, consequently, high metallic properties [14]. These measures improve their conductive properties, enabling them to be subjected to electrical discharge machining and wear-resistance properties to operate in friction pairs (for example, for cutting tools).

**Figure 2:**
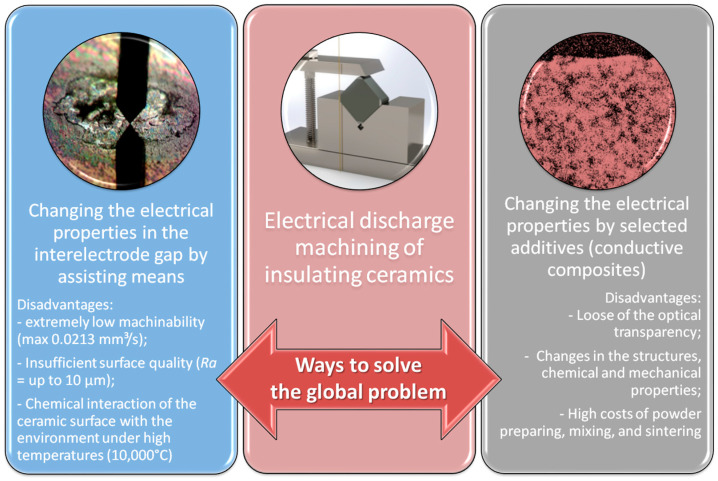
Two main ways to solve the global problem of electrical discharge machining of insulating ceramics and their disadvantages.

The existing techniques of electrical discharge machining insulating ceramics suggest two main methods (Figure 2):

○ By changing the properties of ceramics due to additives selected following the electrical and chemical properties of components [15,16];
○ By changing the electrical and chemical properties in the interelectrode gap [17,18].

The main disadvantage of the first method is the complete and expensive nanomodification of the material with the loss of such properties as optical transparency. The disadvantage of the second method is its low efficiency concerning insulating ceramics, associated with the above-described features. This work aims:

- To review the recent advances and developments in the electrical discharge machining of insulating ceramics;
- To summarize and analyze the current data on the machinability of conductive ceramic composites and the insulating ceramics based on oxides and nitrides in water suspension and hydrocarbons depending on the electrical properties of the ceramics and assisting means;
- To search the approaches and original methods for solving the global problem of the electrical discharge machining of insulating ceramics.

The proposed approach can be extended to develop solutions for electrical discharge machining other insulating materials (including ones filled with glass fiber).

## 2. Electrical Conductivity of Insulating Ceramics and Conductive Composites

Typical ceramics are chemical compounds based on polar bonding. The absence of slip planes makes them extremely brittle by comparison with, for example, steel. When ceramic pieces have been manufactured, they cannot be subjected to heat treatment to induce deliberate changes in their structure. Whereas steels possess a dense structure following crystallization and recrystallization, ceramics fundamentally constitute a multiphase system comprising crystals, a binder phase, and pores [19].

Natural electrical conduction by free lattice electrons makes some carbide, carbonitride, and nitride ceramics electrically conductive (delocalized metal bond *d*-element carbides of IV–X groups excluding platinum group metals). For insulating ceramics (mainly oxide ceramics and nitride ceramics of some elements of III–V groups such as AlN), two mechanisms are utilized to make engineering ceramics electrically conductive:

- Doping with conductive elements;
- Incorporating impurity atoms [20].

Electrically conductive carbides, carbonitrides, and nitrides serve as usual doping components to make insulating ceramics electrically conductive. These ceramics are primarily used as doping components because they are chemically neutral to oxygen. It should be noted that other nitrides mainly exhibit unstable chemically active and explosive properties (Cu_3_N, Mg_3_N_2_, Pb_3_N_2_, Zn_3_N_2_) and decomposes to oxides.

The electrical conductivity of oxide or nitride ceramic composites can be improved by combining the transition-metal carbide or nitride particles with the electrical conductivity of ρ = 10^2^–10^6^ µΩ·cm and the high strength and wear resistance of the matrix [21]. The improved high-performance ceramic matrix presents a fine-grained microstructure (Table 1) that does not deteriorate to operating temperatures close to 1000 °C. The Vickers hardness and fracture toughness of the composites increased with TiC phase addition into Al_2_O_3_-TiC composites containing 30 and 40 vol.% TiC. The composite with 40 vol.% TiC showed the highest flexural strength (687 ± 39 MPa), fracture toughness (7.8 ± 0.4 MPa·m^1/2^), and hardness (22.3 ± 0.3 GPa) with a homogeneous distribution of the second phase within the ceramic matrix [22]. The conductive properties mainly depend on conductive additives concentration [23] when the mechanical properties do not correlate with the content of the ceramic basis (Al_2_O_3_-content, Table 1).

The conductors have specific electrical resistance ρ < 10^−5^ Ω·m as the specific electrical resistance of dielectrics is ρ > 10⁸ Ω·m. The value of specific electrical resistance for produced conductive ceramic with 30 vol.% of TiC is close to the value for carbon in the form of graphite (ρ = 8 × 10^−6^ Ω·m) [28]. Thus, the samples of sintered Al_2_O_3_ + TiC ceramic with more than 30 vol.% of TiC are suitable for electrical discharge machining [25,26,27]. The electrical conductivity of some conductors can fluctuate in the range of 10–10^−12^ S/m (ρ = 10^−1^–10^12^ Ω·m) and also depends on many factors, including temperature and crystal lattice orientation (some materials, such as carbon, exhibit anisotropy of electrical properties) [29,30,31].

The addition of carbon nanotubes (CNTs) to the zirconia matrix in zirconia-based composites induces a substantial increase in electrical conductivity associated primarily with the fact that zirconium and zirconium carbide formed because of the thermochemical reaction have similar electrical conductivity (Table 2) [24,32,33,34,35]. The formation of zirconium carbide occurs through the thermal dissociation of zirconium dioxide and the reduction of lower oxides with carbon:

(1) $${\mathrm{ZrO}}_{2}+H_{2}\overset{>T_{1}}{\to }\mathrm{Zr}+H_{2}O$$

(2) $$2\mathrm{Zr}+O_{2}\overset{T_{2}}{\to }2\mathrm{ZrO}$$

(3) $$\mathrm{ZrO}+C\overset{\sim 2273K}{\to }\mathrm{ZrC}+\mathrm{CO}↑$$

where T_1_ and T_2_ are the subjects of a separate study.

**Figure 3:**
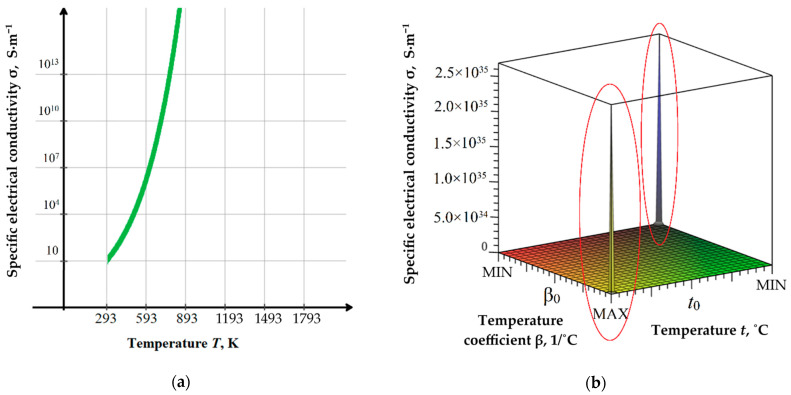
Theoretical specific electrical conductivity σ for dielectric and semiconductive oxide and nitride ceramics (by the example of ZrO_2_): (**a**) with β = *const*, where *t*_0_ = +20 °C and σ_0_ = 10 S·m^−1^; (**b**) with the theoretically varied the temperature coefficient β, where *t*_0_ = +20 °C and σ_0_ = 10^−8^ S·m^−1^, extremes (marked red) may correspond to superconducting conditions.

Simultaneously, it provokes relatively slight thermal conductivity changes (Table 2). The specific electrical conductivity of amorphous oxide ceramics and semiconductors increases significantly with an increase in temperatures since the concentration and mobility of charge carriers are exponentially dependent on temperature (*t*, °C) as was approximated:

(4) $$\sigma \;={\;\sigma }_{0}\cdot e^{\beta \cdot t},$$

where σ_0_ is the value of specific electrical conductivity at normal temperature, and *β* is the temperature coefficient (1/°C). It can be presented as follows (Figure 3). As can be seen with ρ > 10^8^ Ω·m and variable value *β* (for different materials), it is possible to achieve superconductivity conditions at the lowest and highest temperatures. The graphs for other types of dielectric and semiconductive oxide and nitride ceramics have similar trends and vary in σ_0_. For crystalline ceramics, the observed increase in specific and volumetric electrical conductivity is less pronounced, and for metals, it has the opposite tendency [24,39].

An introduction of 30–40 vol. % TiN*_x_* (*x* is in the range of 0.6–1.2; electrical resistivity ρ at +20 °C of 1.0 × 10^−7^–4.0 × 10^−7^ Ω·m in bulk; 3.0 × 10^−7^–1.0 × 10^−6^ Ω·m by plasma vapor deposition (PVD); 2.0 × 10^−6^–1.0 × 10^−4^ Ω·m by chemical vapor deposition (CVD)) into Si_3_N_4_ (1.0 × 10^4^–1.0 × 10^13^ Ω·m) provides an electrically conductive composite with a decreased electrical resistivity of ~10^−5^ Ω·m. Introducing the conductive component also improves mechanical properties such as fracture toughness, strength, and wear resistance [40,41,42].

The problem with Al-based ceramics is in forming pyrophoric ionic salt-like carbides Al_3_C_4_ or Al_2_(C_2_)_3_ (yellow crystals), where Al_3_C_4_ is methanide with (C = C)^4−^ and Al_2_(C_2_)_3_ is acetylenide with (C = C)^2−^ that exhibit insulating properties as for Be_2_C, Li_4_C, Mg_2_C (C^4−^) or Ag_2_C_2_, CuC_2_, CaC_2_, Li_2_C_2_, BaC_2_ (C_2_^2−^) unlike metal-like carbides (Cr*_x_*C*_y_*, Fe*_x_*C*_y_*, Mo*_x_*C*_y_*, Nb*_x_*C*_y_*, Ni*_x_*C*_y_*, Ta*_x_*C*_y_*, TiC, V*_x_*C*_y_*, WC). At a temperature of the thermal decomposition (dissociation), Al_2_O_3_ ceramics dissociate as follows [24,43]:

(5) $$2{\mathrm{Al}}_{2}O_{3}\overset{>3173K}{\to }4\mathrm{Al}+3O_{2}↑$$

(6) $$4\mathrm{Al}+3C\overset{\mathrm{direct}\;\mathrm{reaction}}{\to }{\mathrm{Al}}_{4}C_{3}$$

or

(7) $$2{\mathrm{Al}}_{2}O_{3}+9C\overset{>3173K}{\to }{\mathrm{Al}}_{4}C_{3}+6\mathrm{CO}↑$$

Electrical erosion of alumina in the absence of hydrocarbons can be accompanied by a chemical reaction of dissociated molecules that compose the following insoluble sediment, which decomposes when heated above 848 K:

(8) $$2{\mathrm{Al}}_{2}O_{3}+6H_{2}O\overset{>848K}{\to }4\mathrm{Al}{\left(\mathrm{OH}\right)}_{3}↓$$

A similar issue is related to the dissociation of AlN:

(9) $$2\mathrm{AlN}\overset{>1253}{\to }2\mathrm{Al}+N_{2}↑$$

The released aluminum reacts with the carbon similarly to (5). It can hydrolyze slowly in water:

(10) $$\mathrm{AlN}+3H_{2}O\to \mathrm{Al}{\left(\mathrm{OH}\right)}_{3}↓+{\mathrm{NH}}_{3}↑$$

At the atmosphere, nanofilms of alumina with a thickness of several nanometers are formed at temperatures above 973 K, which can keep from hydrolysis to temperatures above 1643 K. Above this temperature, bulk oxidation occurs:

(11) $$4\mathrm{AlN}+3O_{2}↑\overset{>973K}{\to }2{\mathrm{Al}}_{2}O_{3}+2N_{2}↑$$

## 3. Advances in Electrical Discharge Machining of Ceramics

### 3.1. Electrical Discharge Machining of Conductive Ceramic Composites

**Figure 4:**
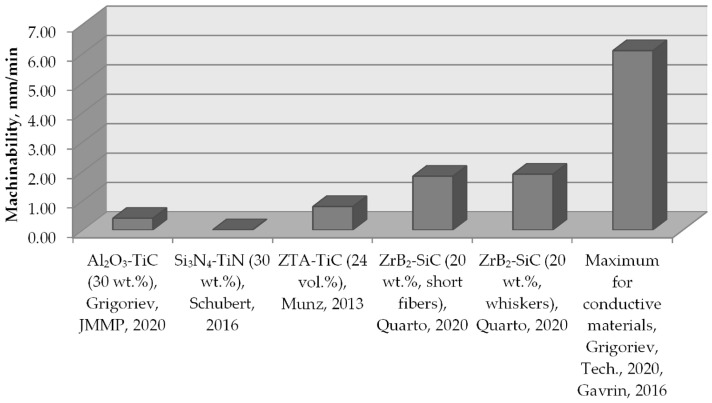
The graphical presentation of conductive ceramic composites’ machinability via electrical discharge machining comparing with the maximal achievable machinability for the conductive materials, where Grigoriev, JMMP, 2020 [11], Schubert, 2016 [45], Munz, 2013 [46], Quarto, 2020 [47], Grigoriev, Tech., 2020 [48], Gavrin, 2016 [49].

The machinability of conductive ceramic composites in electrical discharge machining is presented in Table 3. A graphical presentation of the data compared with the maximal achievable machinability for the conductive materials (aluminum alloys, pure chrome, and copper) is shown in Figure 4.

In [26], it was shown that at 30 vol.% of TiC in Al_2_O_3_-TiC composite, the electrical conductivity corresponds to the adequate level that exceeds the percolation threshold, while Al_2_O_3_-TiC composite exhibits conductivity close to the conductivity of pure titanium at 40 vol.% of TiC. These types of ceramic composites have been known since the mid-1970s [29,30,31]. Still, many research groups conduct experiments on improving their properties by experimenting with technological factors and the dispersion of precursors.

The authors of [44] propose electrically conductive homogenous and dense SiAlON-TiN (10 wt.% and 20 wt.%) ceramic nanocomposite produced by advanced spark plasma sintering at 1700 °C suitable for wire electrical discharge machining. The nanocomposite with 20 wt.% TiN showed specific electrical resistance ρ lower than 1–3 Ω·m.

**Figure 5:**
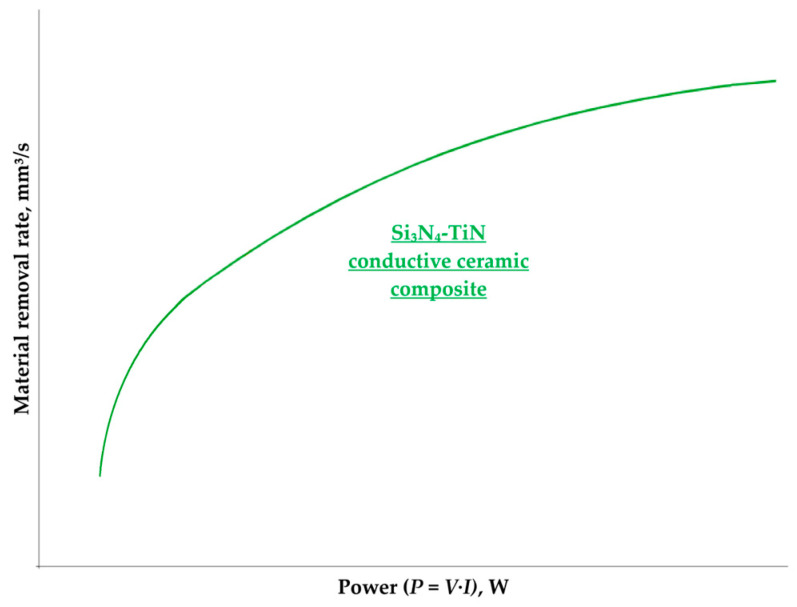
The dependence of the machinability on power for Si_3_N_4_-TiN conductive ceramics in electrical discharge milling (logarithmic regression), based on [45].

A. Schubert et al. experimentally investigated electrical discharge milling (ED milling) parameters of titanium nitride toughened silicon nitride (Si_3_N_4_-TiN) [45]. As Si_3_N_4_-TiN is decomposed at a high temperature in the discharge gap, it exhibits a particular ablation behavior that allows machining with a material removal rate of 200% compared to insulating ceramics of Al_2_O_3_-ZrO_2_ type. The maximum bore depth was 605 μm. The material removal rate was increased by increasing the power (Figure 5). A volume fraction of 30% of the conductive TiN phase is above the percolation threshold, and the electrical conductivity is provided by the TiN network [50]. For hot-pressed Si_3_N_4_-TiN (30vol.%), lowering the sintering temperature from 1700 to 1500 °C decreases resistivity from 0.18 to 0.004 Ω cm. The percolation threshold (*V*_c_) is determined as follows:

(12) $$V_{c}=100\cdot \left[\frac{1}{1+\left(\frac{4}{\theta }\cdot \frac{D_{i}}{d_{c}}\right)}\right],$$

where θ is a quantity of the conductive particles on the surface of the insulating ones; *D_i_* and *d_c_* are the diameters of the insulating and conductive particles. A higher diameter ratio (*D_i_*/*d_c_*) results in a lower *V_c_*. The decomposition of the conductive ceramic composites unavoidably leads to a porous and foamy microstructure shown in [11] and correlates with analytic results of plasma chemistry [51]. During machining in oil, Si_3_N_4_-TiN decomposes on conductive carbides (TiC, SiC), and alumina–zirconia forms conductive ZrC [47] and non-conductive pyrotechnically active Al_3_C_4_ [52,53]. The formed Al_3_C_4_ can actively hamper processing by changing the discharge gap’s electrical conditions since hydrocarbon oil exhibits better electrical conductivity (1.9–4.3 Ω۔m depending on oil purity) than insulating ceramics (≤10^−4^ Ω۔m) [11].

M. Munz et al. [46] added TiC to zirconia and investigated the electrical discharge drilling of ZTA-TiC ceramics. They studied material removal rate, discharge energy, pulse shapes, peak current, and discharge time. They observed that the triangle pulse with a current of 20 A or less is better than the rectangle pulse for the electrical discharge drilling.

The suitable factors to accomplish the electrical discharge machining of conductive ceramic composites are the following:

- Assisting electrode is not required;
- Operational current of 0.05 A [26], 20 A or less [45];
- Operational voltage varies from 20 and 120 to 270 V [26];
- Frequency is 5 kHz;
- Discharge gap depends on the electrical conductivity of the workpiece material and is 10–200 µm.

### 3.2. Electrical Discharge Machining of Insulating Ceramics

**Figure 6:**
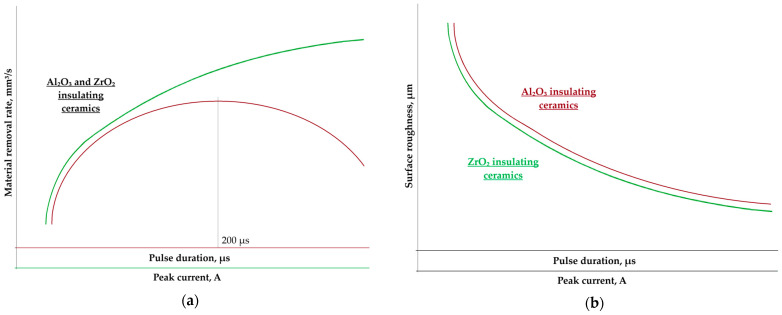
The dependence of the machinability on peak current (logarithmic regression) and pulse duration (quadratic regression or exponential attenuation) (**a**); the dependence of the surface roughness on peak current and pulse duration (logarithmic regression) (**b**) for Al_2_O_3_ and ZrO_2_ insulating ceramics in electrical discharge machining, based on [56].

D. Hanaoka et al. mentioned that the electrical conductivity limit was below 4 × 10^−2^ S·m^−1^ [54]. The machining speed increases as the peak current and pulse width increase. The removal mechanism changes as the pulse width increases. As analyzed from the experiments, when the pulse width was set as 20–30 μs, the machining was stable, and a smoother machined surface was obtained. The pulse interval is not the dominant factor in increasing the machining speed but in improving the machined surface accuracy [55]. The material removal rate of Al_2_O_3_ and ZrO_2_ using adherent copper foil rose with an increase in peak current and pulse duration (Figure 6a). Additionally, the electrode wear rate of Al_2_O_3_ and ZrO_2_ increased with peak current but reduced with pulse duration [56]. However, when the pulse duration exceeded 200 μs, the tool wear rate during the Al_2_O_3_ ceramic machining demonstrated the reverse trend: the tool wear rate slightly increased at a pulse duration longer than 200 μs. The surface roughness of Al_2_O_3_ and ZrO_2_ increased with peak current and pulse duration (Figure 6b). Moreover, the surface roughness of the Al_2_O_3_ ceramic after electrical discharge machining was higher than for the ZrO_2_ ceramic at identical machining conditions that correlated with the plasma chemical nature of the processes between electrodes in the presence of heat higher than 3500 °C, which remained unexplained in the paper. The surface integrities of ZrO_2_ revealed more machining debris adhered to the machined surface, and the Al_2_O_3_ machined surface and thermal spalling features were rougher than for ZrO_2_. In general, the machining performance (material removal and tool wear rates) of ZrO_2_ with the copper adherent tape is better than Al_2_O_3_ with the same adherent. H. Zhang et al. [57] developed a technique to provide pulse generators with self-adapting voltage-adjusting for the electrical discharge machining of Al_2_O_3_ insulating ceramic. They demonstrated that the pulse generator could improve energy availability and discharge flexibility.

N. Ojha et al. [58] monitored the electrical signals when machining copper and non-conductive ceramic Si_3_N_4_. Voltage and current sparks’ signals were shown. They demonstrated that the most remarkable differences were a significant decrease for Si_3_N_4_ in the ringing behavior of the voltage signal and the absence of reverse current for Si_3_N_4_, and a decrease in the peak current value detected in the case of Si_3_N_4_. H. Gotoh et al. [59] used a unique technique for machining insulating ceramics Si_3_N_4_ with electrical discharge milling. They used a method in which the pulse discharge was longer than the pulse duration. Hence, the electrically conductive layer is created on the insulating ceramic surface, enabling machining.

**Figure 7:**
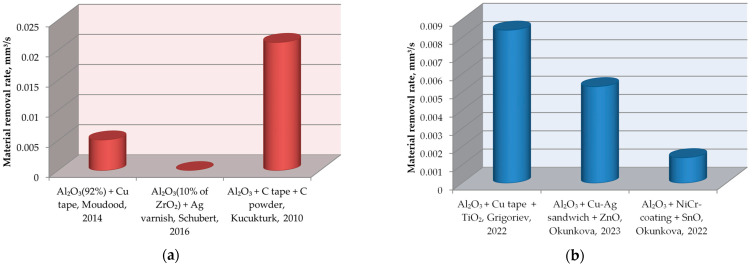
The graphical presentation of Al_2_O_3_ insulating ceramic machinability via electrical discharge machining: (**a**) in hydrocarbons, (**b**) in water suspension, where Schubert, 2016 [45], Moudood, 2014 [63], Grigoriev, 2022 [65], Kucukturk, 2010 [66], Okunkova, 2023 [67], Okunkova, 2022 [68].

The results of applying the assisting electrode technique primarily depend on the method of pre-coating the insulating ceramic workpiece and the main properties of the film (adhesion, thickness, chemical composition) (Table 4). The graphical presentation of the machinability of Al_2_O_3_ insulating ceramics is shown in Figure 7. As seen, the machinability in oil is 2.54 times higher than in water. From all the used assisting means, copper tape in combination with TiO_2_ powder provides higher machinability in the water-based dielectric medium. After processing, the film can be chemically removed from the ceramic surface with acetone, K_2_CrO_4_-H_2_SO_4_ mixture, or other solvents [60,61].

There are several types of coating to accomplish the process, such as copper assisting electrode [56,62,63,64,65], silver varnish [45,64], carbon conductive layer [66], multimaterial sandwich coatings [64,67], and Ni-Cr coating by plasma vapor deposition [68]. The assisting electrode made of copper tape can be removed easily from the AlN [64], Al_2_O_3_ [65], and ZrO_2_ [69] ceramic surface after the machining. There are also examples of electrical discharge machining of Si_3_N_4_ ceramics in TiO_2_ water-based suspension using conductive TiN coating deposited by plasma vapor deposition [70]. Any plasma vapor deposition coatings are difficult to remove after machining due to diffusion between the insulating workpiece and coating materials. However, the authors propose using assisting coating as a multifunctional one that provides conductive properties to the surface layer of the insulating workpiece and is used as a wear-resistant coating to prolongate the operational life of the Si_3_N_4_ ceramic cutting insert. In [66], it was experimentally shown that machinability strongly depends on electrical resistivity for TiO_2_, SiC, and ZrO_2_ ceramics during electrical discharge drilling when there is probably other nature (obviously, thermochemical combining with electrophysical) in Al_2_O_3_ and SiO_2_ ceramics machining, but the trends are similar.

The authors that research the electrical discharge machining of ZrO_2_ ceramics in hydrocarbon oil [56,66,71,72] report on forming conductive ZrC with the same conductivity as pure Zr [24]. Electrical discharge machining of aluminum-containing insulating ceramics in oil presents an unsolved scientific problem related to the high electronegativity of aluminum, which is very active towards oxygen and forms non-conductive carbides in its absence that are mistakenly named pyrolitic carbon. In [66], it was reported that not only “pyrolytic carbon” was identified in the working area.

A typical crater-like surface was detected during the electrical discharge machining of ZrO_2_ ceramics [45]. Thermal cracks and cleavages characterize the AlN ceramic surface [64]. The Al_2_O_3_ ceramic surface [68] is characterized by the traces of the thermal sublimation and drop-like deposition of the secondary structure of the compositions of secondary order formed by the products of the interaction of the decomposition of ceramics, assisting powder and dielectric medium.

The authors [64] consider chemical and plasma vapor deposition methods to be time-consuming and require costly equipment compared with the technique of AlN ceramics coating with the labor-intensive two-layered Ag sandwich coating with Ag nanoparticles between them additionally covered with copper tape. However, the PVD technique has demonstrated impressive results with a material removal rate of 0.0014 mm^3^/s in machining Al_2_O_3_ ceramics [68], which is more than two times higher than 0.006 mm^3^/s for AlN ceramics [64]. The actual problem of any plasma deposition technique of copper and silver coating is related to droplet formation [73,74] and coating low adhesion to insulating ceramics with a coating thickness above 3–4 µm [75,76]. At the same time, there is a global problem of discharge impulse reinitiating in the working zone when most of the coating is sublimated and decomposed in the dielectric medium. In this context, the Ni-Cr coating [68] has shown better prospects: it has smoother surface characterization when deposited on the insulating ceramic surface, exhibits better adhesion at the coating thickness up to 15 µm, and it can form intermetallic compounds of Al*_x_*Ni*_y_* type (when *x* = 1, 3, … and *y* = 1, 3, …) [77] during coating sublimation and decomposition. These types of compounds exhibit superconductive properties and have prospects for future development. It should be noted that the addition of silver nanoparticles in the concentration of 0.1 g/L at the working zone fundamentally changes the electrical condition in the interelectrode gap [64]. It improves the material removal rate by ~6 times compared to triple copper tape and by ~30 times compared to silver double coating sandwich with Ag nanopowder.

The authors of [66] noticed that the programmed depth faster was achieved with a thicker conductive layer in a hydrocarbon medium. However, the authors reported that they did not understand the processes’ chemical nature during the electrical discharge machining of Al_2_O_3_ and ZrO_2_ ceramics, which needs additional research and explanation from the thermo- and electrochemistry point of view.

It is noticed that during electrical discharge machining of Si_3_N_4_ insulating ceramics, carbon-containing substances of the kerosene under high temperatures are often cracked and stuck to the positive pole in the voltage field [17]. The electrically conductive material adhered to the insulating workpiece’s surface during the electrical discharge machining of Si_3_N_4_, ZrO_2_ in kerosene. It consisted of decomposable carbon-containing substances from the fluid that can be conductive carbides of the ZrC group and semiconductive SiC adhered to the insulating workpiece surface. An electrically conductive carbide layer was formed during continuously generated electrical discharges. Thus, insulating ceramics are transformed into electrically machinable material during electrical discharge machining. However, during the electrical discharge machining of the sintered Al_2_O_3_ + 20% of ZrO_2_ stabilized with 3.9% of Y_2_O_3_ in kerosene, the conductive layer was not specified [78], obviously due to the known insulating properties of Al_3_C_4_. The eroded layer was removed mechanically after electrical discharge machining.

A standard silver varnish with 45% silver content was used to create the conductive layer of approximately 20 μm on the surface of ZrO_2_ ceramics [71]. After drying, the layer formation’s uniformity was tested by measuring the resistance using a Fluke multimeter. When the first layer was machined, a conducting rebuilt layer was generated on the workpiece, visible as black surface areas. The detailed composition of this layer was not under investigation. Still, it can be proposed that due to the thermal decomposition of the hydrocarbon oil by the electrical discharges and following plasma channel, ZrC was formed.

With an assisting electrode applied by a screen printing when electrical discharge machining of a semi-conductive silicon carbide [79] that did not exhibit electrical conductivity, the process was initially unstable, and it required the adjustment of machining factors such as current and frequency and tool geometry. The authors found that the cause of unstable machining might be the excessive generation of carbonized products. The average thickness of the carbonized layer on the ceramic surface was 24 µm with a standard deviation of 1.6 µm. The adaptation of tool geometry was produced to improve flushing in the working area. The maximum machined depth was 420 µm. The maximum material removal rate was 3.58 × 10^−3^ mm^3^/min (~0.215 mm^3^/s). Detached microstructures with an aspect ratio of 30 are machined. The present material removal mechanism was indicated as thermally induced spalling. No heat-affected zone was detected. The same group of researchers studied the electrical discharge machining of SiC-based composites [80]. The assisting electrode was generated from the decomposed dielectric oil and deposited on the ceramic surface. It is found that the conductivity of the SiC-based materials does not influence the machinability of ceramics’ microtexturing.

The suitable factors to accomplish the electrical discharge machining of insulating ceramics are the following:

- Assisting electrode material—copper tape/foil, silver coating/varnish, carbon tape, Cu + Ag sandwich, Ni + Cr PVD-coating;
- Assisting electrode thickness—from 12 to 300 µm;
- Type of assisting electrode deposition—adhesive, diffusion;
- Assisting powder material—Ag nanoparticles, graphite, SnO, TiO_2_, ZnO;
- Assisting powder granule size—from nano size to 10–30 µm in diameter;
- Assisting powder concentration for suspension of 0.1–150 g/L;
- Operational current of 0.3–0.4 A;
- Operational voltage of 108 V;
- The frequency of 2, 5, 7, 10, 30 kHz;
- Pulse duration of 0.5–2.5 µs;
- The discharge gap of 48–60 µm.

## 4. Discussion

Let us represent the summarized data on machinability via EDM presented in Table 3 and Table 4 in the form of graphs to approximate the obtained graphs up to the closest functions and analyze the revealed dependencies.

**Figure 8:**
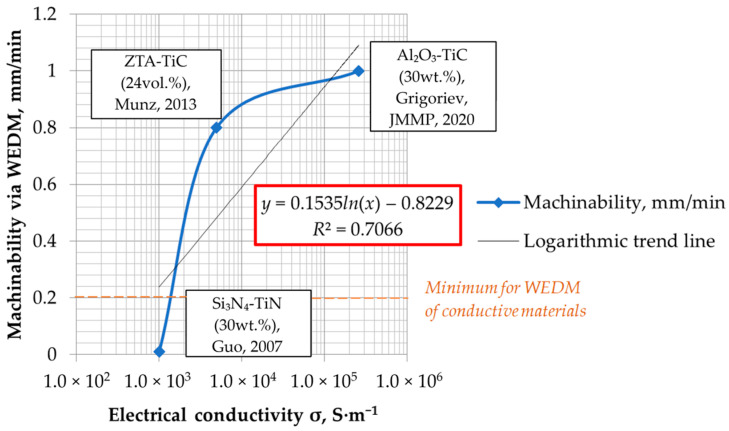
The dependence of conductive ceramic composites’ machinability via wire electrical discharge machining on electrical conductivity, where *R*^2^ is a coefficient of determination, Grigoriev, JMMP, 2020 [11], Munz, 2013 [46], Guo, 2007 [50].

The dependence of conductive ceramic composites’ machinability via electrical discharge machining on electrical conductivity for some conductive ceramic composites based on Table 3 is presented in Figure 8. As can be seen, the dependence has a character of the logarithmic regression with a coefficient of determination of 0.7066:

The higher the electrical conductivity, the higher the machinability.

**Figure 9:**
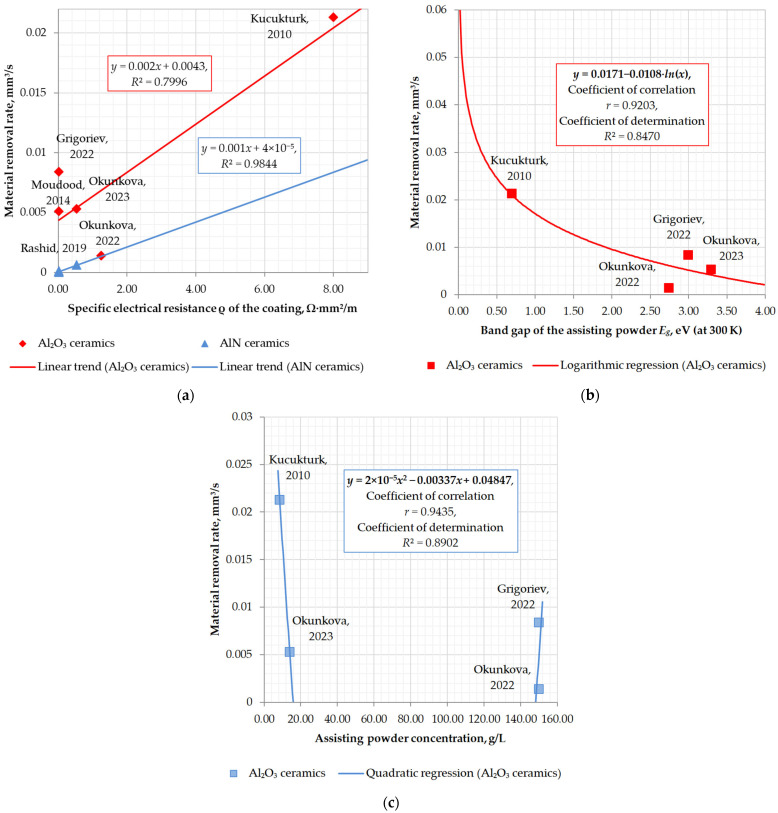
The graphical presentation of Al-containing insulating ceramic machinability via electrical discharge machining: (**a**) dependence on the specific electrical resistance of the assisting coating; (**b**) dependence on the band gap of the assisting powder; (**c**) dependence on the assisting powder concentration, where Moudood, 2014 [63], Rashid, 2019 [64], Grigoriev, 2022 [65], Kucukturk, 2010 [66], Okunkova, 2023 [67], Okunkova, 2022 [68].

Figure 9 presents graphs based on data from Table 4 depending on the specific resistance of the assisting coatings, the band gap, and the concentration of the assisting powder. The data are provided for Al_2_O_3_ and AlN ceramics. As it is seen in Figure 9a, the material removal rate is in inverse relationship with the specific resistance of the assisting coatings, and it is evident that despite the conviction of many authors that it is the electrical properties of the assisting coating that play a key role in the machinability of insulating ceramics. There should be the influence of other factors previously overlooked by the authors when developing a research plan and selecting an assisting electrode material. Figure 9b demonstrates the exponential relationship between the material removal rate and the band gap of the assisting powder for Al_2_O_3_ ceramics machined in hydrocarbons and water suspension (as more band gap is, as more dielectric properties exhibit material, up to 3–4 eV for semiconductors and more than 4–5 eV for dielectrics). Thus, the machinability dependence on the assisting powder’s electrical properties is proven:

The lower the band gap, the higher the machinability.

The graph presents the results of the approximation. That is a logarithmic regression of the following type:

(13) $$y=y_{0}\cdot \left(b-a\cdot ln\left(x\right)\right),$$

where *a* and *b* are empirical coefficients and require additional research. The data presented in Figure 9c are correlated with the data obtained in [65,67], where the assisting powder concentration influence on the material removal rate was researched in the concentration gap of 50–150 g/L and 7–100 g/L, correspondingly. The most pronounced results were achieved at 14 and 150 g/L. The reduction in machinability was observed in the range of the powder concentration from 21 to 100 g/L. The dependence corresponds to the quadratic regression as follows:

(14) $$y=y_{0}\cdot \left(a\cdot x^{2}+b\cdot x+c\right)$$

and can be a part of the logarithmic decrement of attenuation of the following type:

(15) $$y=y_{0}\cdot e^{-a\cdot x}$$

that seems more relevant to the mathematical expression of physical phenomena and requires more study.

For further development, it should be noted that TiO_2_ and ZnO are semiconductive with a band gap of 3.00–3.26 eV for TiO_2_ depending on its form (for anatase is more than for rutile) and ~3.37 eV for ZnO. It can be quite a perspective to consider adding other semiconductors with lower band gap values to improve conductivity conditions in the interelectrode gap. However, most of them as Cd_3_As_2_, Cd_3_P_2_, SnTe, VO_2_, Zn_3_P_2_ (the band gap of ~0, 0.5, 0.18, 0.7, 1.5 eV correspondingly [81,82,83,84,85]) are highly poisonous (4 points for health by NFPA 704). This excludes any possibility of their development and uses in the conditions of real production in the future. However, two of them deserve attention for further consideration—copper sulfides (Cu*_x_*S*_y_*) and rare Tin(II) sulfide (α-SnS) with band energies of ~1.2 and 1.0–1.3 eV, correspondingly. They are available on the market in powder form.

Solving the problems of electrical discharge machining application techniques for aluminum-containing ceramics is of fundamental importance for science and applications such as electric power, the space industry, optics, and a promising cutting tool made of cutting ceramics. The development of superhard and highly wear- and heat-resistant materials based on ceramics with

○ A low coefficient of linear thermal expansion (α × 10^6^ of 7.0–8.0 K^−1^ for Al_2_O_3_, 4.0–6.0 K^−1^ for AlN, 3.0 K^−1^ for SiAlON at 20–1000 °C);
○ Low thermal conductivity at normal temperatures (λ of 22–25 W·(m·K)^−1^ for Al_2_O_3_, > 170 W·(m·K)^−1^ for AlN, 85 W·(m·K)^−1^ for SiAlON at 20–100 °C); and
○ Excellent optical properties (spectral transmission range of 0.17–5.5 µm for Al_2_O_3_, 0.2–5.0 µm for thin films of AlN [86], 2.5–5.0 for SiAlON [87]) by volumetric shaping will make it possible to create a group of new classes of metal-cutting tools with exceptional exploitation properties.

These tools can be aimed at machining high-entropy alloys, such as nickel- and titanium-based alloys achieving 800–1000 °C in the contact zone in friction pair [70,88], and carry out the sixth technological transition associated with Kondratieff’s waves [89,90].

The better results for the electrical discharge machining of insulating ceramics using assisting means can be summarized as follows (Table 5).

## 5. Conclusions

The conducted review allowed the recent advances in the electrical discharge machining of insulating ceramics to be summarized and analyzed, and revealed the dependences of the machinability on:

○ The electrical properties of the ceramics for conductive ceramic composites (logarithmic regression);
○ The specific electrical resistance of the assisting coating (needs additional research), the band gap (logarithmic regression) and the powder concentration of the assisting powder (quadratic regression) for insulating ceramics.

It has been shown that the lower the band gap, the higher the machinability. There are summarized data on electrical discharge machining factors for conductive ceramic composites and insulating ceramics.

The proposed approach of using the semiconductive powder composition with the small band gap can be extended to develop solutions for electrical discharge machining other insulating materials.

The electrical discharge machining of insulating ceramics has vast potential. It requires transitioning from laboratory conditions to real industrial applications to process reinforcing ceramics and other new ceramic-based composites to produce:

- A novel helical cutting tool with exploitation properties that can be of a critical advantage in milling new nickel- and titan-based and superalloys above 800 °C close to or even exceeding the properties of the pure sintered oxide and nitride ceramics;
- A new nanocomposite with physical properties that exceed the properties of the known materials in electrical resistivity, hot hardness, resistance to abrasive wear, linear thermal expansion, and thermal conductivity that can be not available to be shaped by traditional milling and turning due to their exceptional properties for aerospace applications.

The existing technological approaches in electrical discharge machining insulating ceramics have no advantages or disadvantages. The technology itself is not suitable for machining insulating materials and, by the existing logic, should not provide any results. However, some researchers put their efforts into exploring the limits of the existing technology of electrical discharge machining without putting any mechanical effort on the surface of the insulating materials using special measures such as providing an electrically conductive layer on the surface of the insulating ceramics. The machining is performed using the thermal effect of the electrical discharges, providing conductive particles and/or debris in the discharge gap to make the discharges denser and changing the electrical conditions in the interelectrode gap. The most outstanding in this context is the fact that many research groups achieve the machinability of the insulating ceramics by electrical discharge machining that is much less than for the convenient conductive materials but still shows impressive data (up to 0.0051–0.0213 mm^3^/s).

## Figures and Tables

**Table 1:** Electrophysical and mechanical properties of Al_2_O_3_-based ceramics.

Type of Ceramics	Specific Electrical Resistance ρ at +20 °C, Ω·mm^2^/m	Flexural Strength, MPa	Fracture Toughness, MPa·m^1/2^	Vickers Hardness, GPa	Average Particle Size, µm	Reference
Al_2_O_3_ (99.8 wt.%)	10^12^–10^14^	630	4.3	20.00	Not provided	[23,24]
Al_2_O_3_ (99 wt.%)	Not provided	500	3.5	18.65	3.0–4.0	[25]
Al_2_O_3_ (85 vol.%)-SiC (15 vol.%)	Not provided	850	6.5	22.00	1.0–2.0	[25]
Al_2_O_3_ (70 vol.%)-TiC-TiN (22.5 vol.% of TiC + 7.5 vol.% of TiN)	Not provided	890	5.6	21.80	0.14–1.0	[23]
Al_2_O_3_ (70 vol.%)-TiC (30 vol.%)	3.382	643 ± 48	7.1 ± 0.3	22.80 ± 0.30	0.53–0.6	[26]
Al_2_O_3_ (70 vol.%)-TiN (30 vol.%, Cortinitis)	Not provided	640	4.5	18.65–20.00	2.0	[25,27]
Al_2_O_3_ (60 vol.%)-TiC-TiN (10 vol.% of TiC_µm_ + 10 vol. of TiC_nm_ + 20 vol.% of TiN)	Not provided	881.4	7.8	20.80	0.14–1.0	[23]
Al_2_O_3_ (60 vol.%)-TiC (29.85 vol.% of TiC + 0.5 vol.% of MgO)	Not provided	630	5.2	17.80	1.39	[28]
Al_2_O_3_ (60 vol.%)-TiC (40 vol.%)	Not provided	600	4.2	20.00	2.0–3.0	[25]
Al_2_O_3_ (60 vol.%)-TiC (40 vol.%)	0.589	687 ± 39	7.8 ± 0.4	23.30 ± 0.30	0.53–0.6	[26]
Al_2_O_3_ (60 vol.%)-TiC (40 vol.%, commercial sample)	3.510	637	3–5	13.50–14.00	Not provided	[26]
Al_2_O_3_ (40 vol.%)-TiC (55 vol.% of TiC + 2.25 vol.% of Mo + 2.25 vol.% of Ni)	Not provided	890	7.8	Not provided	0.5	[22]
Al_2_O_3_ (35 vol.%)-TiC (55 vol.% of TiC_µm_ + 5 vol.% of TiC_nm_ + 2.25 vol.% of Mo + 2.25 vol.% of Ni)	Not provided	840	7.5	Not provided	0.04	[22]

**Table 2:** Electrical properties of some Zr-containing materials.

Material	Electrical Conductivity σ, S·m^−1^	Specific Electrical Resistance ρ at +20 °C, Ω·m	Specific Electrical Resistance ρ * at +20 °C, Ω·mm^2^/m	Thermal Conductivity α at +20 °C, W·m^−1^·K^−1^	Reference
ZrO_2_ † (Insulator)	10^−6^–10^−12^	10^−6^–10^12^	10^8^–10^14^	1.7–2.7	[32,33,34]
ZrO_2_ + 0.5 wt.% CNT ‡ (Insulator)	1.0 × 10^1^	1.0 × 10^−1^	1.0 × 10^1^	3.3 §	[35]
ZrO_2_ + 1 wt.% CNT (Insulator)	3.16 × 10^2^	3.16 × 10^−3^	3.16 × 10^−1^	2.8 §	[35]
ZrO_2_ + 2 wt.% CNT (Insulator)	7.50 × 10^2^	1.33 × 10^−3^	1.33 × 10^−1^	Not provided	[35]
ZrO_2_ + 3 wt.% CNT (Insulator)	8.66 × 10^2^	1.15 × 10^−3^	1.15 × 10^−1^	Not provided	[35]
ZrO_2_ + 4 wt.% CNT (Insulator)	1.0 × 10^3^	1.0 × 10^−3^	1.0 × 10^−1^	2.6 §	[35]
ZrC † (Conductor)	2.0 × 10^6^	5.0 × 10^−7^	5.0 × 10^−5^	11.6	[36,37]
Zr † (Conductor)	2.27 × 10^6^	4.41 × 10^−7^	4.41 × 10^−5^	22.7	[38]

* calculated for convenience; † given for references; ‡ CNT is carbon nanotubes; § obtained by spark plasma sintering at 1350 °C.

**Table 3:** Machinability of conductive ceramic composites in electrical discharge machining.

Material	Workpiece Thickness/Machined Depth, mm	Electrical Conductivity σ, S·m^−1^	Technology of Producing	Electrode Tool	Main EDM Factors	Dielectric Medium	Machinability	Reference
Al_2_O_3_-TiC (30 wt.%)	10	2.60 × 10^5^ (3.77 Ω·mm^2^/m)	WEDM	Brass wire, Ø250 µm	*V*_0_ = 108 V; *f* = 5 kHz; *D* = 1 µs	Deionized water, no flushing	0.4 mm/min (feed rate, no obstacles)	[11]
Al_2_O_3_-TiC (30 wt.%)	2–4	2.96 × 10^5^	WEDM	Brass wire, Ø250 µm	*V*_0_ = 270 V; *I_e_* = 0.05 A	Deionized water with conductivity of 0.1 µS/cm, no flushing	Suitable for EDM	[26]
Al_2_O_3_-TiC (40 wt.%)		1.70 × 10^6^					Suitable for EDM	[26]
SiAlON-TiN (10 wt.%)	3	9.80 × 10^−3^ * (1.02 × 10^2^ Ω·m)	WEDM	Brass wire, Ø250 µm	*V*_0_ = 72 V; *I_e_* = 1.9 A; *D* = 2 µs (relaxation)	Deionized water with conductivity of 0.1 µS/cm, no flushing	Suitable for EDM	[44]
SiAlON-TiN (20 wt.%)		6.25 × 10^3^ * (1.6 × 10^−4^ Ω·m)					Suitable for EDM	[44]
Si_3_N_4_-TiN (~30 wt.% *)	0.605 (× ø0.123 mm, programmed depth of 0.5)	Did not exhibit any (coated with Ag varnish of 20 µm), calculated circuit linear resistance of 4.78 × 10^5^ Ω/m	Micro-EDM (ED drilling)	WC rod (6% cobalt binder) of ø115 µm	*V*_0_ = 165, 180 V; *I_e_* = 70 A; *D* = 6 µs	Hydrocarbon oil (HEDMA-111)	0.010–0.011 mm/min for 58 min of machining or 7.76 × 10^−6^ mm^3^/s † for an area of 0.045 mm^2^	[45]
ZTA-TiC (alumina + 17 vol.% of zirconia, 1.5 vol.% of yttria, 24 vol.% of TiC)	10	4.93 × 10^3^	ED drilling	Copper tube electrode (Typ-D) of ø2000 µm	*V*_0_ = 130 V; *I_e_* = 15, 20 A; *D* = 40, 60 µs	Oil-based fluid with flushing 1–6 L/h	Theoretical feed rate of ~0.8–1.0 mm/min (active feed rate/proportion of effective pulses)	[46]
ZrB_2_-SiC (20 wt.%, short fibers)	0.2 (× ø1.0 mm)	Not provided	Micro-EDM (ED drilling)	WC rod electrode of ø300 µm	*V*_0_ = 70 V; *I_e_* = 29 A; *D* = 0.7 µs	Hydrocarbon oil	6.14 × 10^−8^ mm^3^ per discharge (18.26 mm/min for an area of 0.28 mm^2^) ‡	[47]
ZrB_2_-SiC (20 wt.%, whiskers)		Not provided			*V*_0_ = 71 V; *I_e_* = 29 A; *D* = 0.7 µs		6.38 × 10^−8^ mm^3^ per discharge (18.97 mm/min for an area of 0.28 mm^2^) ‡	[47]

*V*_0_ is the operational voltage; *f* is the frequency; *D* is the pulse duration; *I_e_* is the operational current; * experimentally obtained; † calculated for convenience; ‡ it should be noted that the conventional material removal rate for wire electrical discharge machining (WEDM) of conductive materials is 0.2–12 mm/min [48], but practice dictates that in most of the cases it is 0.8–1.0 mm/min, and maximum for aluminum is about 4–6 mm/min [49], and it is highly possible that authors have been mistaken in their calculations or did not take into account electrode relaxation time in discharge timing and the actual linear material removal rate should not exceed 1.82 and 1.90 mm/min, correspondingly.

**Table 4:** Types of assisting electrodes for electrical discharge machining of aluminum-containing insulating ceramics.

Assisting Electrode Material	Assisting Electrode Shape/Thickness, µm	Type of Deposition	Primary Electrode Material and Shape	Technology	Dielectric Medium	Insulating Workpiece Material	Maximum Depth, µm	Machining Time, min	Material Removal Rate, mm^3^/s	Reference
Copper	Foil/Not provided	Adhesive	Not provided	EDM	Kerosene	Al_2_O_3_, ZrO_2_	Not provided	Not provided	Not provided	[56]
	Sheet/Not provided	Not provided	Steel	ED Milling	Water-based emulsion	Al_2_O_3_	Not provided	Not provided	Not provided	[62]
	Foil/60 µm	Adhesive	Cu, 5000 × 5000 µm	EDM	Kerosene	Al_2_O_3_ (92% of purity)	1500	~120 * (37.5 mm^3^/0.0051 mm^3^/s = 7352 s)	0.0051	[63]
	Triple layered copper tape/150 µm	Adhesive	Cu-W, Ø600 µm	Micro-EDM	Hydrocarbon (mineral) oil, no flushing, no rotation	AlN	700 (from tape surface)	120	0.0001 * (0.79 mm^3^/7200 s)	[64]
	Copper tape/40 µm	Adhesive	Brass wire, Ø250 µm	WEDM	Deionized water + TiO_2_ particles of Ø10 µm (150 g/L)	Al_2_O_3_	52 × 111 × 7250 (D × W × L)	0.17 (10 s)	0.0084	[65]
Silver	Varnish (45% of Ag) applied with a Paintbrush/20 µm	Adhesive	A WC rod with 6% of Co binder, Ø115 or 500 µm †	Micro-EDM (proposed for ED Milling)	Hydrocarbon (mineral) oil HEDMA-111	ATZ ceramic composite (10% of Al_2_O_3_ toughened ZrO_2_)	731 × Ø120 (for programmed depth of 500 µm)	43	Not provided	[45]
	Double paint layer of Ag coating with Ag nanopowder in between/Not provided	Diffusion, baked sandwich at 900 °C for 60 min	Cu-W, Ø600 µm	Micro-EDM	Hydrocarbon (mineral) oil, no flushing, no rotation	AlN	700 (from coating surface)	600	0.00002 * (0.79 mm^3^/36,000 s)	[64]
Carbon	Polymer-based material containing carbon powder/Not provided	Adhesive, heated at 150 °C	Cu pipe of Ø3500 µm with a hole of Ø3000 µm	EDM	Kerosene with graphite powder of Ø30 µm (7–10 g/L), flushing at 1.5 bar pressure	Al_2_O_3_	~5000	~235	0.0213	[66]
Ag + Cu	Ag nanoparticles sandwiched between two paint layers of Ag coating with Cu tape on top/Not provided	Diffusion, baked sandwich at 900 °C for 60 min	Cu-W, Ø308 µm	Micro-EDM	Hydrocarbon (mineral) oil with Ag nanoparticle (0.1 g/L), no flushing, no rotation	AlN	1693 (from coating surface)	150	0.0006 * (0.50 mm^3^/9000 s)	[64]
	Cu-Ag doubled-layered coating (Cu-tape + polymer glue with Ag-powder)/300 µm	Adhesive	Brass wire, Ø250 µm	WEDM	Deionized water + ZnO particles of Ø10 µm (14 g/L)	Al_2_O_3_	980 (from workpiece surface)	Not provided	0.0032–0.0053	[67]
Ni + Cr	Ni-Cr PVD coating/12 µm	Diffusion	Brass wire, Ø250 µm	WEDM	Deionized water + SnO particles of Ø10 µm (150 g/L)	Al_2_O_3_	50 × 320 × 4000 (D × W × L)	0.5 (30 s)	0.0014	[68]

* calculated for convenience; † the authors provide two different values in two paragraphs.

**Table 5:** The summarized results of electrical discharge machining of insulating ceramics.

Insulating Ceramics	Assisting Electrode (Coating) Material/Type of Deposition	Assisting Electrode Thickness, µm	Dielectric Medium	Assisting Powder	Primary Electrode Material and Shape	Adhered Material at the Primary Electrode	Theoretical Chemical Composition of the Assisting Debris [24,39]	Material Removal rate, mm^3^/s	Reference
Al_2_O_3_	Copper tape/adhesive	40	Water-based suspension	TiO_2_ particles of Ø10 µm (150 g/L)	Brass wire, Ø250 µm	Not reported	Cu^2+^ (conductive)	0.0084	[65]
	Ni-Cr coating/diffusion	12		SnO particles of Ø10 µm (150 g/L)	Brass wire, Ø250 µm	Not reported	Al*_x_*Ni*_y_* (conductive)	0.014	[68]
AlN	Ag nanoparticles sandwiched between two layers of Ag varnish with Cu tape on top/adhesive	Not provided	Hydrocarbon oil, no flushing, no rotation	Ag nanoparticle (0.1 g/L)	Cu-W, Ø308 µm	Not reported	Ag^1+^ (conductive)	0.0006 *	[64]
Si_3_N_4_	Not provided (expected TiN coating/diffusion based on [18]) †	Not provided	Kerosene oil, rotation at 100 min^−1^	Not provided	Cu, ø2000 µm	SiC (semi-conductive)	SiC (semi-conductive)/TiC (conductive)/TiN (conductive)	~0.51–0.84	[59]
TiO_2_	Carbon tape (polymer-based)/adhesive	Not provided	Kerosene, flushing at 1.5 bar pressure	Graphite powder of Ø30 µm (7–10 g/L)	Cu pipe of Ø3500 µm with a hole of Ø3000 µm	TiC (conductive)	TiC (conductive)	~0.45	[66]
ZrO_2_						ZrC (conductive)	ZrC (conductive)	~0.55	

* calculated for convenience; † the authors did not provide data on the conductive layer but have mentioned using it; based on similar works [18], it can be concluded that TiN coating deposited by PVD was used.

## Data Availability

Data are available in a publicly accessible repository.
